# Direct monitoring of the stepwise condensation of kinetoplast DNA networks

**DOI:** 10.1038/s41598-021-81045-6

**Published:** 2021-01-15

**Authors:** Nurit Yaffe, Dvir Rotem, Awakash Soni, Danny Porath, Joseph Shlomai

**Affiliations:** 1 Department of Microbiology and Molecular Genetics, Institute of Medical Research Israel-Canada, The Kuvin Center for the Study of Infectious and Tropical Diseases, The Hebrew University-Hadassah Medical School, 91120 Jerusalem, Israel; 2 Institute of Chemistry, The Center for Nanoscience and Nanotechnology, Edmond J. Safra Campus, The Hebrew University of Jerusalem, 91904 Jerusalem, Israel

**Keywords:** DNA, DNA metabolism

## Abstract

Condensation and remodeling of nuclear genomes play an essential role in the regulation of gene expression and replication. Yet, our understanding of these processes and their regulatory role in other DNA-containing organelles, has been limited. This study focuses on the packaging of kinetoplast DNA (kDNA), the mitochondrial genome of kinetoplastids. Severe tropical diseases, affecting large human populations and livestock, are caused by pathogenic species of this group of protists. kDNA consists of several thousand DNA minicircles and several dozen DNA maxicircles that are linked topologically into a remarkable DNA network, which is condensed into a mitochondrial nucleoid. In vitro analyses implicated the replication protein UMSBP in the decondensation of kDNA, which enables the initiation of kDNA replication. Here, we monitored the condensation of kDNA, using fluorescence and atomic force microscopy. Analysis of condensation intermediates revealed that kDNA condensation proceeds via sequential hierarchical steps, where multiple interconnected local condensation foci are generated and further assemble into higher order condensation centers, leading to complete condensation of the network. This process is also affected by the maxicircles component of kDNA. The structure of condensing kDNA intermediates sheds light on the structural organization of the condensed kDNA network within the mitochondrial nucleoid.

## Introduction

Tropical diseases, such as African sleeping sickness, South and Central American Chagas disease and the Leishmaniases, affecting large human populations and livestock, are caused by infection of parasitic protists of the group Trypanosomatidae. A unique feature, shared by all trypanosomatid species, is their remarkable mitochondrial genome, known as kinetoplast DNA (kDNA), which has an unusual structure and genetic function. In the species *Crithidia fasciculata*, used in the current study, kDNA consists of ~ 5,000 duplex DNA minicircles of 2.5 Kbp each and ~ 25 maxicircles of 37 Kbp^1–4^, which are topologically linked. Minicircles within the network are topologically relaxed^5^ and each minicircle is linked on average to three other minicircles, yielding a network with a valence of 3 in a honeycomb arrangement, where each minicircle is typically at the vertex of a hexagonal cell^6–8^.

Packaging of genomes has been observed in cells and DNA-containing organelles, in which genomes are found as intricate nucleoprotein complexes, forming the nucleoid structure in bacteria^14,15^ and in eukaryotic subcellular organelles, such as mitochondria^16,17^ and chloroplasts^18,19^, as well as in the nucleosome-based chromatin structure in the eukaryotic nucleus^20,21^. Condensation of genomes limits their accessibility to the replication and transcription machineries. This has been studied extensively in the case of nuclear chromatin, revealing that uncoiling of the nucleosomal complex, known as chromatin remodeling, is mediated mainly by histone posttranslational modifications, which decrease their affinity to DNA^22^.

**Figure 1:**
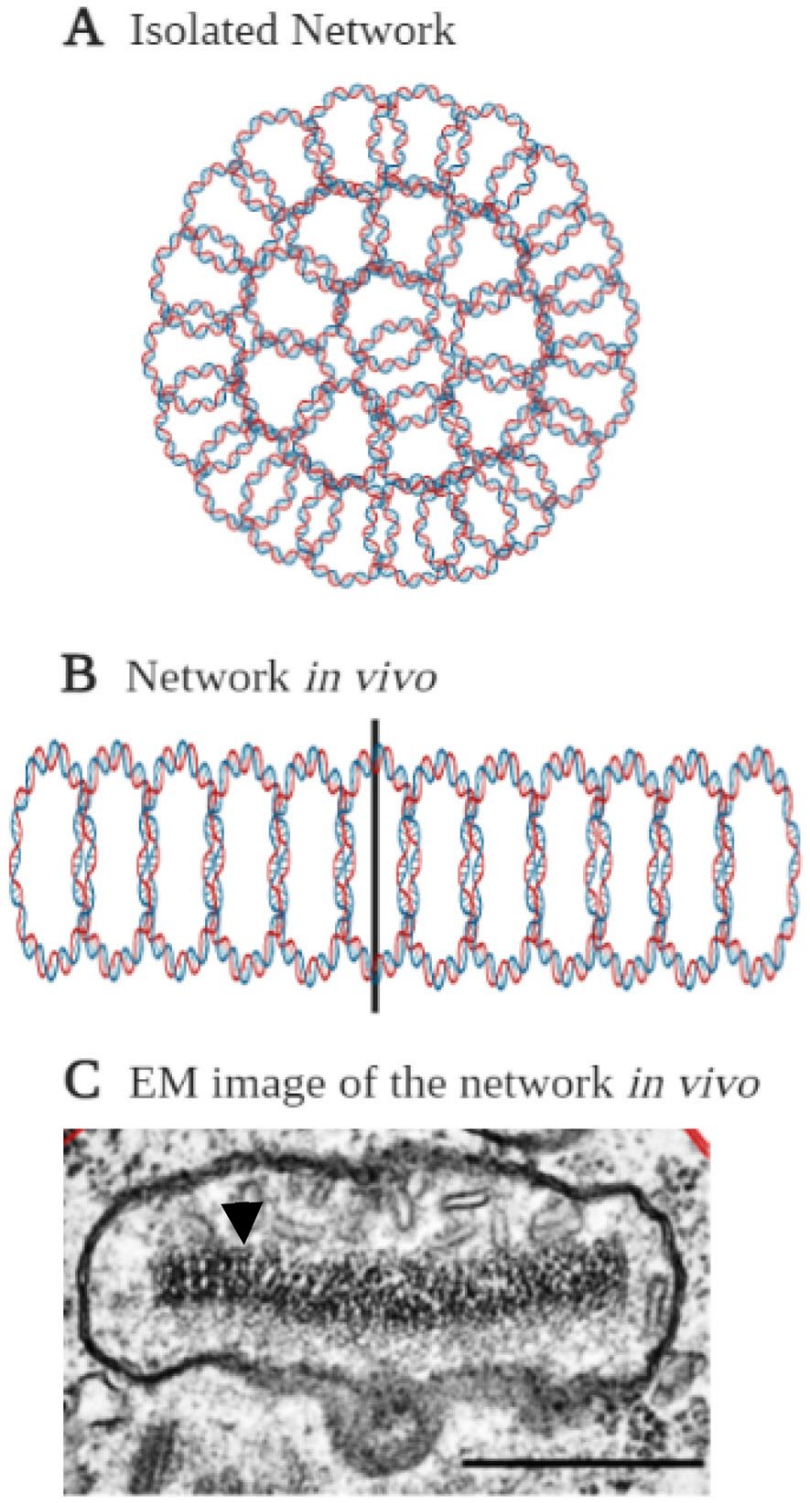
Organization of the kDNA network. **(A)** Diagram of an isolated, uncondensed kDNA network, in which minicircles lie flat in the plane. **(B,C)** Condensed kDNA networks in vivo. **(B)** Diagram of a section of kDNA network, condensed in vivo into a disc-shape structure of 1.0 × 0.4 mm, in which the interlocked minicircles are stretched out and stand side by side. The vertical bar represents the axis of the disc. The height of the disc is approximately one half the circumference of a minicircle (0.4 µm). **(C)** An electron micrograph of a thin section of a *T. brucei* cell, focusing on its single mitochondrion. The black arrowhead marks the condensed kDNA disc, within the mitochondrial matrix. Bar, 500 nm. **(A,B)** were created in BioRender (https://biorender.com/).

kDNA is a good model system for studying the packaging of DNA. Electron microscopy (EM) analyses revealed that isolated kDNA networks form an elliptical cup-shaped structure, with a major axis of 15 µm and a minor axis of 10 µm^9^. As suggested by these analyses, the kDNA network condenses in vivo into a disc structure of 1.0 × 0.4 µm^10,11^, in which the interlocked minicircles are stretched out and stand side by side. The height of the disc is approximately half the circumference of a minicircle (0.4 µm). Hence, the disc consists of rows (stacks) of minicircles and its width is determined by the stretch-out minicircles (for illustration see Fig. 1) Four mitochondrial proteins, known as kinetoplast-associated proteins (KAP1-4), have previously been studied in *C. fasciculata*, and implicated with the condensation and compaction of the kDNA network into a disc-shaped nucleoid in the mitochondrial matrix^12–17^. KAPs are small basic proteins, which resemble the nuclear histone H1 protein in their lysine and alanine-rich domains and their preferential and highly cooperative binding to DNA containing oligo(dA)-oligo(dT) tracts^13,18^. KAPs were shown to condense kDNA networks in vitro into a tight compact structure^12,13^. Expression of each of these proteins in an *E. coli* mutant deficient of HU protein (a histone-like protein from *E. coli*) rescued a defect of chromosomal condensation and segregation of the mutant cells and restored the compactness of their nucleoids^13^. Wang et al.^19^ have identified an HMG box-containing mitochondrial protein, TbKAP6, in the related organism *Trypanosoma brucei*, which condenses kDNA in vitro and is involved in kDNA minicircle replication. Its depletion by RNAi arrests cell growth and causes the disorganization, shrinkage and loss of kDNA. More recently, de Souza et al.^20^ have described KAP7 in symbiont-harboring trypanosomatids.

We have previously reported^21^ that the in vitro remodeling of KAP3/4-condensed kDNA networks, is mediated by specific protein–protein interactions of the kDNA replication initiator protein, universal minicircle sequence binding protein (UMSBP)^22–24^ with KAP3 and KAP4. Condensation of kDNA in vitro by KAP proteins impairs its decatenation by DNA topoisomerase II and the release of free covalently-closed kDNA minicircles, the exclusive templates for minicircle replication initiation. kDNA remodeling by UMSBP enables the network decatenation by DNA topoisomerase. These studies have suggested that UMSBP-KAP3/4 interactions may play an important role in the pre-replication decondensation of kDNA, by enabling the release of minicircles from the network, and thereby the activation of kDNA replication initiation^21^.

Although the dynamics of kDNA packaging and remodeling plays an essential role in the regulation of kDNA replication initiation, our understanding of the process that condenses kDNA to generate the unique structural organization of this mitochondrial nucleoid has been limited. It is yet unknown what is the pattern of kDNA condensation that leads to its specific structural organization in the condensed form and what constrains affect the final structure of the condensed kDNA disc (recently discussed in Ref^8^). As kDNA condensation and decondensation play an essential role in the control of gene expression and replication, their study is essential for understanding the regulation of these processes in the mitochondrial genome of trypanosomatids.

Here, we address these questions by examining the condensation of kDNA, through the analyses of kDNA condensation intermediate networks, generated by the interactions of kDNA with KAP3. We used fluorescence microscopy and atomic force microscopy (AFM), to analyze the progressing intermediate structure of condensing kDNA along the condensation process, at the single network level, in detail and three dimensions. These analyses revealed that condensation of the kDNA network is a stepwise process. It proceeds through an orderly systematic and hierarchical process, in which DNA circles are condensed locally into condensation foci, consisting of minicircles and maxicircles. These foci are distributed throughout the condensing network, forming a network of condensation foci, which are interconnected by DNA fibers. These local condensation foci increase in size and are then assemble into fewer, higher order condensation centers. Our observations also suggest that while condensation of kDNA networks can be performed by its interaction with a variety of basic proteins, kDNA remodeling depends on the capacity of the condensing protein to interact specifically with the replication initiator UMSBP^21^. Altogether, the structure of condensing kDNA described here may shed light on the unique structural organization within the condensed kDNA nucleoid in vivo.

## Results

### UMSBP remodels kDNA networks condensed by KAP3 but not by poly l-lysine

**Figure 2:**
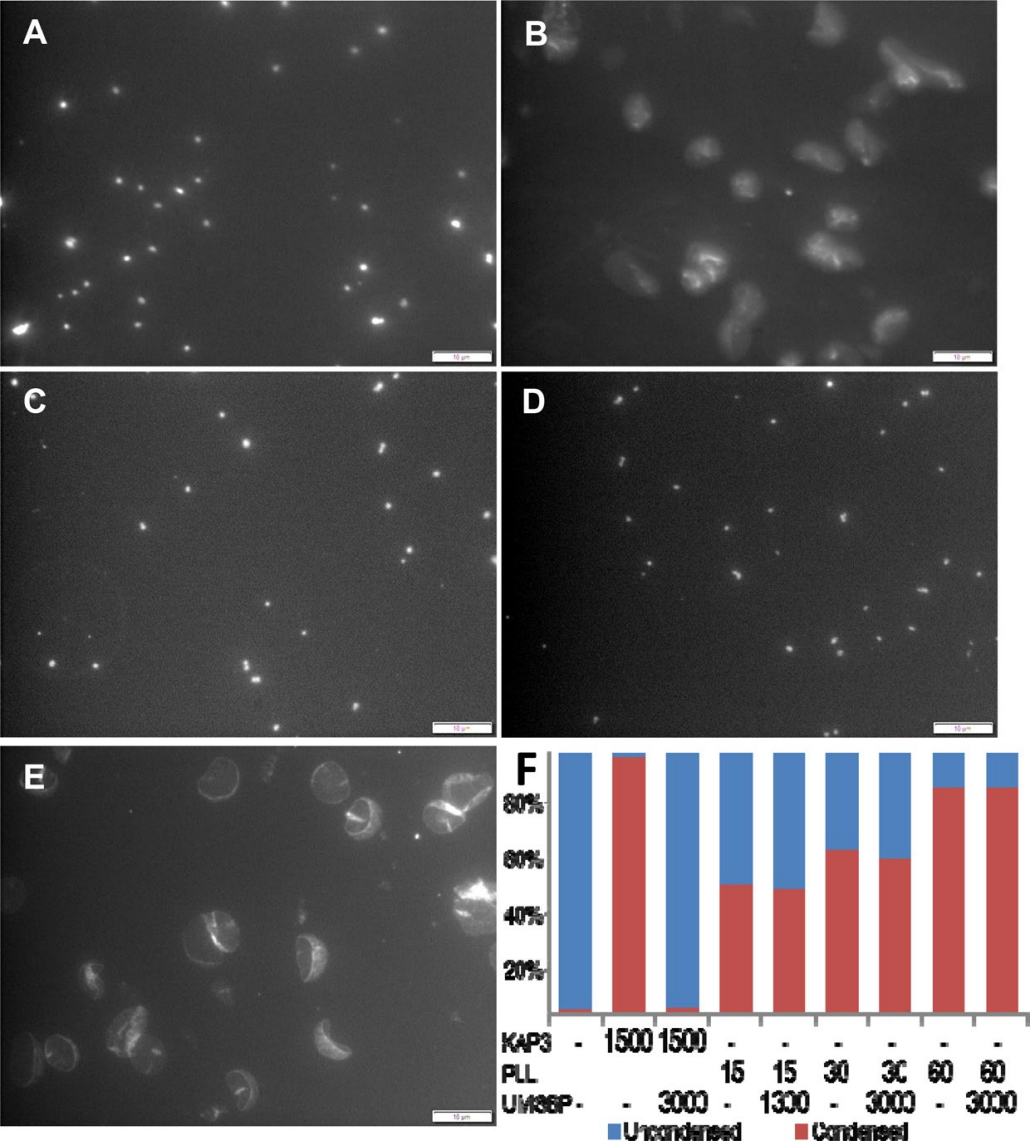
Condensation of kDNA networks by KAP3 and poly L-lysine and their remodeling by UMSBP. DAPI stained isolated kDNA networks, as visualized by fluorescence microscopy, after condensation assays: **(A)** In the presence of 1500 nM KAP3, followed by **(B)** treatment with 3000 nM UMSBP. **(C)** In the presence of 60 nM PLL, followed by **(D)** treatment with 3000 nM UMSBP. **(E)** No protein added. **(F)** The percent of uncondensed and condensed networks was calculated using ~ 600 networks counted for each condensation assay indicated in the plot.

kDNA networks are condensed in vitro by interaction with diverse basic proteins, such as the lysine-rich KAP1-4^13^ and histones H1^21^, as well as by poly l-lysine (Fig. 2). Condensation of kDNA by these basic proteins is mediated mainly by charge neutralization, where only limited affinity of protein to a specific nucleotide sequence in the DNA is displayed. Here, we hypothesize that the specificity of the overall process of kDNA condensation and remodeling, lies in the remodeling reaction. While kDNA condensation, could be mediated through various basic proteins, kDNA decondensation depends on the specific protein–protein interactions of the condensing protein with UMSBP, the remodeling protein that mediates the network decondensation^21^. We have previously suggested^21^ that these protein–protein interactions result in a conformational change in KAP3 and that in its new conformation the protein has a reduced affinity to DNA, resulting in its release from the nucleoprotein complex and the decondensation of DNA. Thus the reason for choosing KAP3 as the kDNA condensing protein in this study was due to its biological relevance to the processes of kDNA condensation-remodeling^21^. As we have previously shown, while each one of the four KAPs of *C. fasciculata* has the capacity to condense kDNA, only KAP3 and KAP4 proteins interact with UMSBP, and these specific protein–protein interactions lead to the remodeling of KAP3/KAP4-condensed kDNA networks^21^. We have also reported that an order of magnitude higher concentration of UMSBP was required, in order to decondense kDNA networks that were condensed by the human histone H1 protein, compared to the UMSBP concentrations needed for decondensation of kDNA networks, condensed by KAP3^21^. To further explore the role of specific protein–protein interactions between UMSBP and the condensing protein, we used here, the simple homo-polypeptide poly l-lysine (PLL), as the kDNA condensing protein. Considering our previous observations using histone H1 protein as kDNA condensing agent^21^, it was anticipated that the impact of UMSBP on the condensation state of a PLL-condensed kDNA would be even more limited. Condensation and remodeling were monitored by quantitative fluorescence microscopy of DAPI stained kDNA networks^12,21^. The results show that interaction of uncondensed kDNA with either KAP3 or PLL resulted in the condensation of kDNA networks (Fig. 2A,C,F). However, while interaction with UMSBP, at two fold molar ratio, resulted in the complete decondensation of the KAP3-condensed networks (Fig. 2B,F), up to two orders of magnitude higher molar excess of UMSBP, failed to decondense the PLL-condensed kDNA (Fig. 2D,F). These observations demonstrated that although poly L-lysine has the capacity to condense kDNA, the PLL-kDNA nucleoprotein complex is inert as a substrate for remodeling by UMSBP, supporting the hypothesis that kDNA decondensation is specific and depends on the capacity of the condensing protein to interact with UMSBP.

### The presence of maxicircles affects the condensation of kDNA networks

**Figure 3:**
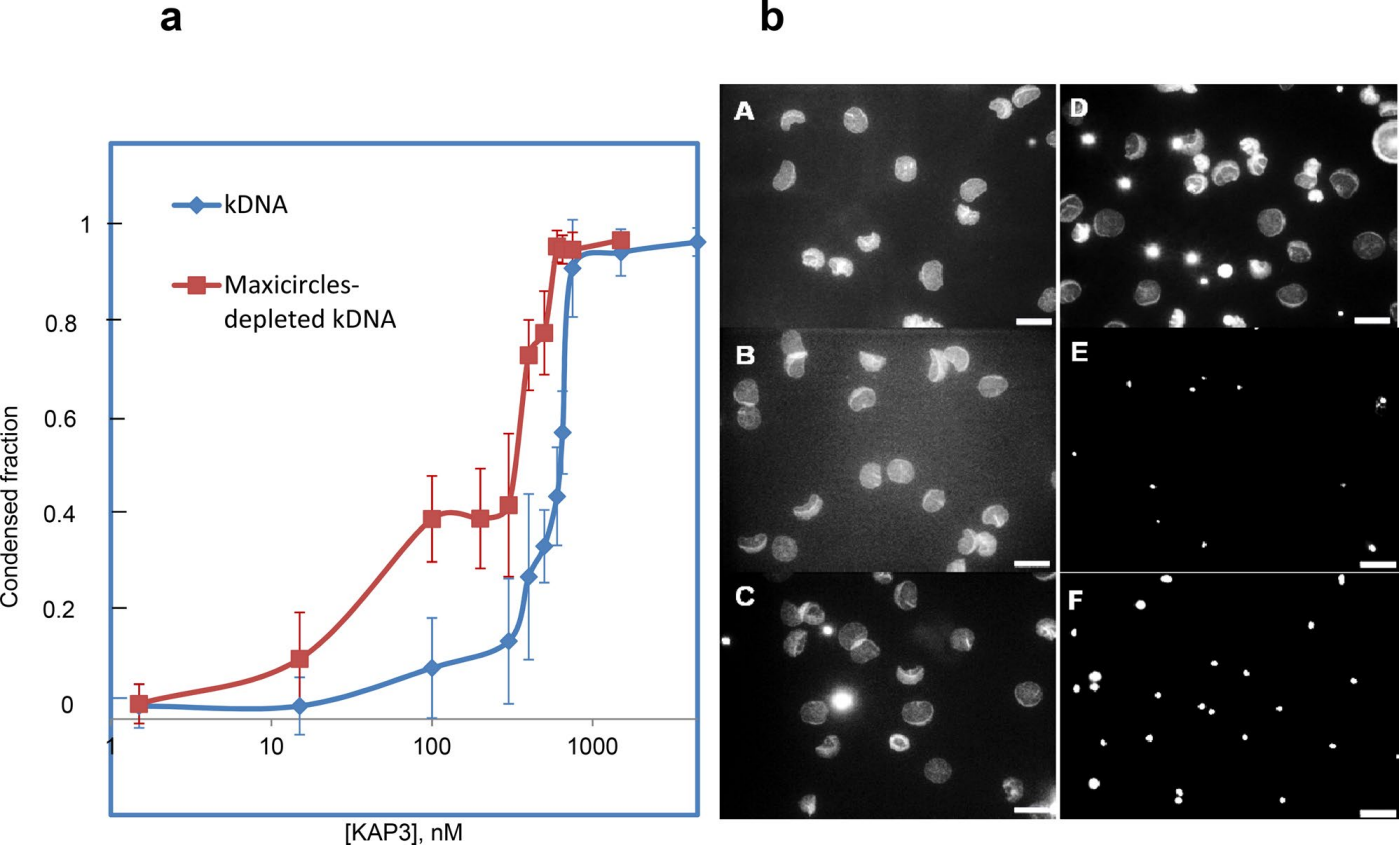
Condensation of kDNA by KAP3. **(a)** Quantification, by fluorescence microscopy, of the condensation of kDNA networks was performed at different KAP3 concentrations (presented on a logarithmic scale) at the range of 1.5–1500 nM, either with native-kDNA networks (blue line) or maxicircles depleted networks (minicircles-kDNA) (red line). Each point in the curves is based on the count of ~ 600 networks. **(b)** A sample of images demonstrating, **(A)** uncondensed kDNA networks; **(B–F)** condensation of kDNA networks by 1.5, 100, 500, 750, 1500 nM KAP3, respectively. Bars are 10 µm.

To monitor the condensation of kDNA networks, we have analyzed kDNA condensation intermediates. A series of kDNA condensation intermediates were generated by reacting uncondensed kDNA networks with increasing concentrations of KAP3. The condensates were first monitored by fluorescence microscopy of DAPI-stained networks (see above, Fig. 2), following Xu and Ray^12^, as we have previously described^21^. Using this assay, products of kDNA-KAP3 interactions were viewed and the abundance of condensed versus uncondensed networks was quantified (Fig. 3) within a KAP3 concentration range of more than three orders of magnitude (1.5 × 10^–9^–4.5 × 10^–6^ M).

Purified kDNA networks, extracted from *C. fasciculata*^21^, consist of a minor fraction (~ 2%) of condensed networks. The abundance of condensed kDNA networks increases gradually in correlation with the increase of KAP3 concentration in the condensation reaction mixture (Fig. 3). The plot of the fraction of condensed kDNA networks versus KAP3 concentration is sigmoidal, indicating the cooperative nature of the interaction, as was previously observed by Xu et al.^13^. It displays three apparent phases, starting with a slow increase (1.5–300 nM KAP3), reaching some saturation at 300 nM. Then, the slope increases sharply beyond 300 nM KAP3, reaching true saturation (94% condensation) at 750 nM KAP3 and 97% at 1.5 µM.

Next, we studied the effect of kDNA components on the network condensation. Each kDNA network consists of two distinct monomeric components, ~ 5000 DNA minicircles and ~ 25 maxicircles, which are interlocked with the minicircles scaffold, but form a distinct topological network in one or more foci within the kDNA network in the different trypanosomatids species^25–27^. To examine the effect of the maxicircle component on the condensation of kDNA, we prepared kDNA networks depleted of maxicircles (herein named ‘minicircles-kDNA’) by specific cleavage using restriction endonuclease *Pst1*^28^, versus networks containing both minicircles and maxicircles (herein named ‘native-kDNA’). The extent of maxicircles depletion in *Pst1-*treated kDNA networks was validated by quantitative PCR, using primers specific to the maxicircle Cox II gene as a DNA template. We used a Real Time PCR protocol to amplify a Cox II amplicon of 513 bp, using both maxicircles-depleted and native kDNA networks. Real Time PCR results, revealed a C_t_ difference = 6, comparing maxicircles-depleted and native kDNA networks, indicating an extensive depletion of maxicircles from these networks (see “Methods” for further details). Minicircles-kDNA networks were used in a condensation reaction, in the presence of increasing concentrations of KAP3, as described above. As shown in Fig. 3, depletion of maxicircles had a significant effect on the kDNA condensation reaction. The sigmoidal curve, indicating the cooperative nature of KAP3-kDNA interaction, is observed also with minicircles-kDNA networks. However, a significantly larger fraction of condensed minicircles-kDNA networks is generated at low concentrations of KAP3. Then, we observed, repeatedly, some saturation (between 100 and 300 nM KAP3), with no increase in the fraction of condensed minicircles-networks. Beyond this KAP3 concentration, the fraction of condensed networks in both the minicircles-kDNA networks and native-kDNA networks increases significantly as a result of relatively small changes in KAP3 concentration. It leads, at 600 nM KAP3, to complete (99%) condensation in minicircles-kDNA networks, yet to a fraction of only 46% condensed native-kDNA networks at this KAP3 concentration.

Altogether, these observations revealed a significant difference (P < 0.05) between the condensation curves of minicircles-kDNA network versus native-networks, in response to their interactions with KAP3. The effect observed upon depletion of maxicircles from kDNA suggested that in maxicircles-depleted kDNA, a lower concentration of KAP3 is required in order to condense a larger fraction of the networks. This may imply that the presence of maxicircles, which are interlocked with minicircles in the native-kDNA network, has a stabilizing effect on the network structure, rendering it less flexible to the structural changes imposed upon the network by its interaction with the condensing protein.

### Early condensation intermediates consist of interconnected foci of DNA circles

The fluorescence microscopy assay described above (Figs. 2 and 3) is of a binary nature. It distinguishes between uncondensed and fully condensed kDNA networks, enabling an estimation of the abundance of these two extreme network species. Yet, the assay has an intrinsic bias, as it cannot distinguish between uncondensed and partially condensed networks, having an apparent similar perimeter, and therefore it overestimates the abundance of uncondensed kDNA networks. Nevertheless, in the presence of a high concentration of KAP3 we noticed the presence of a fraction of the networks population with varying size perimeters. Further analysis of these networks by fluorescence microscopy, revealed the presence of multiple DAPI-stained dense spots within these networks (not shown). We speculated that these dense structures could represent areas (foci) of locally condensed DNA within the partially condensed kDNA networks. However, optical microscopy has not provided the high enough resolution required for analyses of these structures. To characterize the structural changes that take place in condensing kDNA network intermediates in more details and three dimensions, we have used atomic force microscopy (AFM). This method enables the monitoring of structural changes at the nano-scale resolution, with no fixation, staining or metal coating of the networks^11,29^. AFM has been previously used in the study of protein-DNA interactions and DNA dynamics^30–32^, DNA condensation and compaction^33–36^, mitochondrial nucleoids^37^, chromatin structure and remodeling^38–45^, as well as in the study of kDNA structure in *C. fasciculata*^11,20,29,46^, *Trypanosoma Cruzi*^47^, and other kinetoplastids^48^.

**Figure 4:**
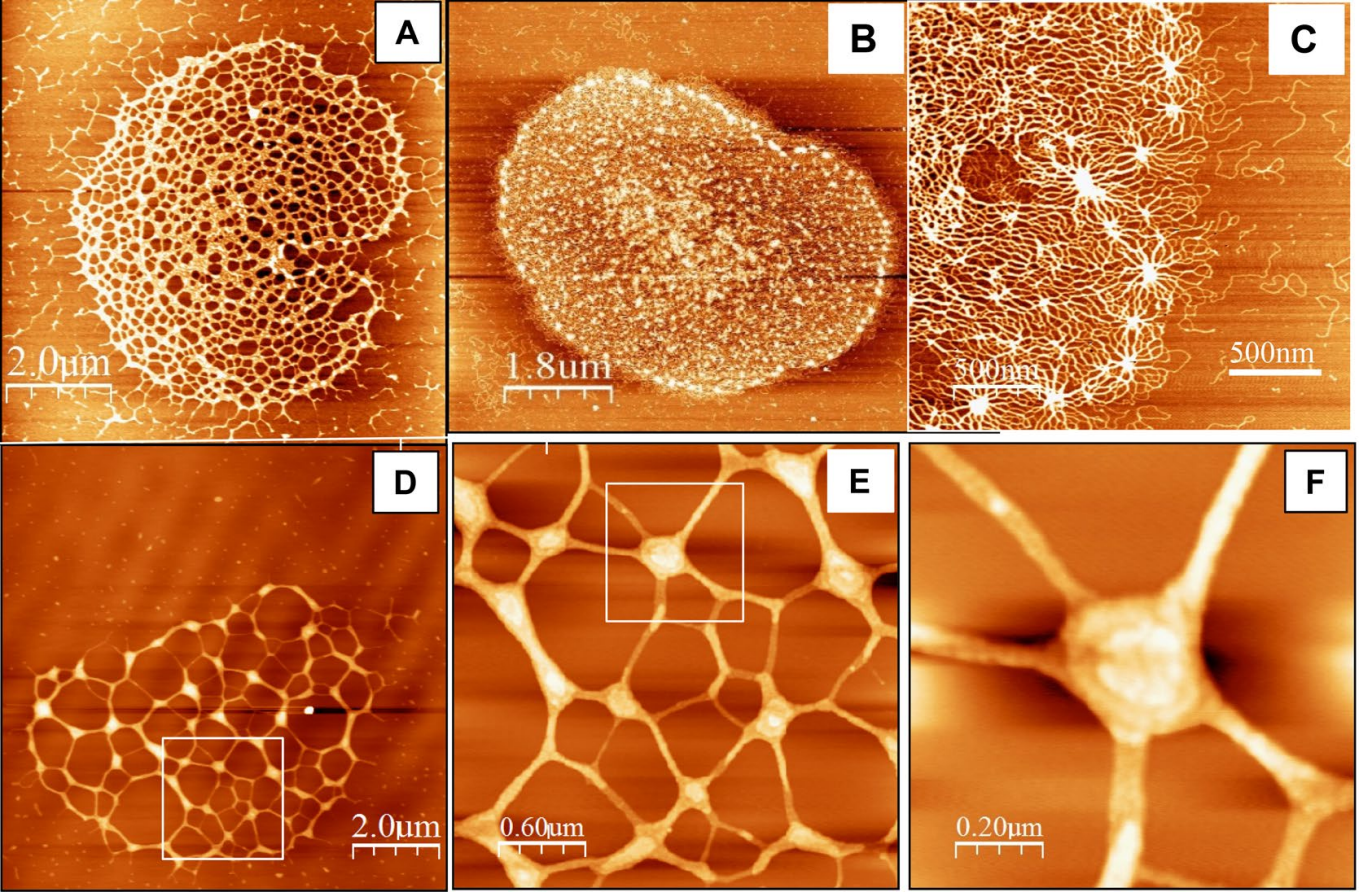
kDNA condensation intermediates consist of interconnected local condensation foci (LCF). Isolation and purification of uncondensed kDNA networks and their analyses by AFM were performed as described in the “Methods”. **(A–C)** Uncondensed kDNA networks (bars = 2.0 µm in **(A)**; 1.8 µm in **(B)**; and 500 nm in **(C)**). **(D)** A kDNA condensation intermediate network resulting from the interaction of an uncondensed kDNA network (5.4 pM) with 1.5 nM KAP3. **(E)** A higher resolution scan of the area defined by a square in the partially condensed network in **(D)**. **(F)** A higher resolution scan of the condensation focus marked by the square in **(E)** (bars in **(D–F)** = 2.0, 0.6 and 0.2 µm, respectively).

**Figure 5:**
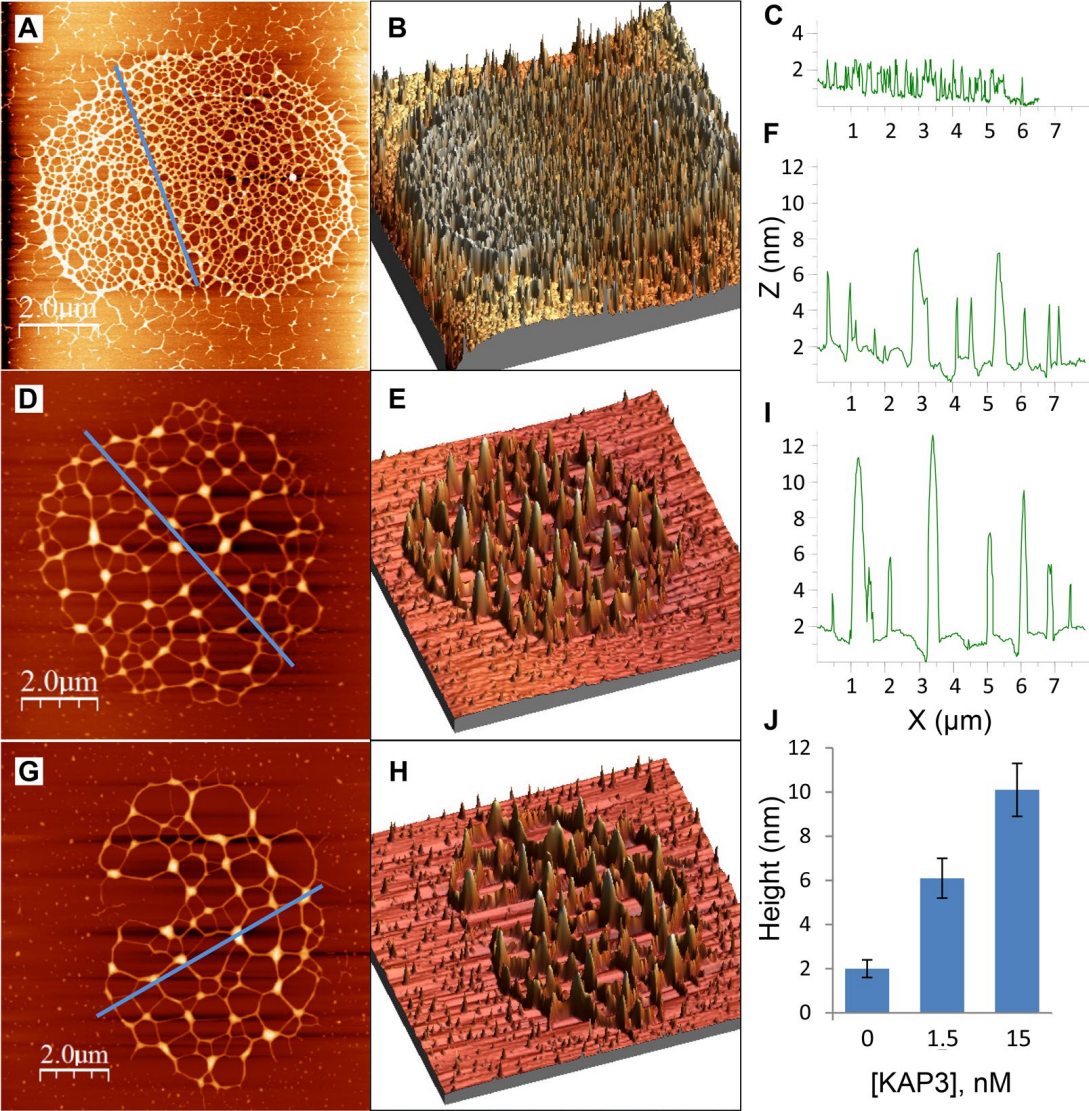
kDNA condensation proceeds via generation of less abundant, larger LCF. **(A,D,G)** Two-dimensional 2D AFM scan images of uncondensed kDNA **(A) **and networks that interacted with 1.5 nM **(D)** and 15 nM **(G)** of KAP3. In **(B,E,H)** Three-dimensional 3D- displays of AFM scans, corresponding to those presented in **(A,D,G)**, respectively. **(C,F,I)** Height profiles of LCF in the AFM scans presented in **(A,D,G)**, respectively, corresponding to the paths indicated by the lines crossing the 2D images. Bars = 2 µm. **(J)** The average heights of LCFs plotted against KAP3 concentrations, as indicated. Three-dimensional 3D-displays of AFM scans were based on the total data collected in the scan. Height profiles in AFM scans, were based on measurements corresponding to the random paths, indicated by the lines crossing the images. Networks analyzed: uncondensed kDNA networks (n = 22), partially condensed networks in the presence of 1.5 nM (n = 17) and 15 nM KAP3 (n = 11).

**Figure 6:**
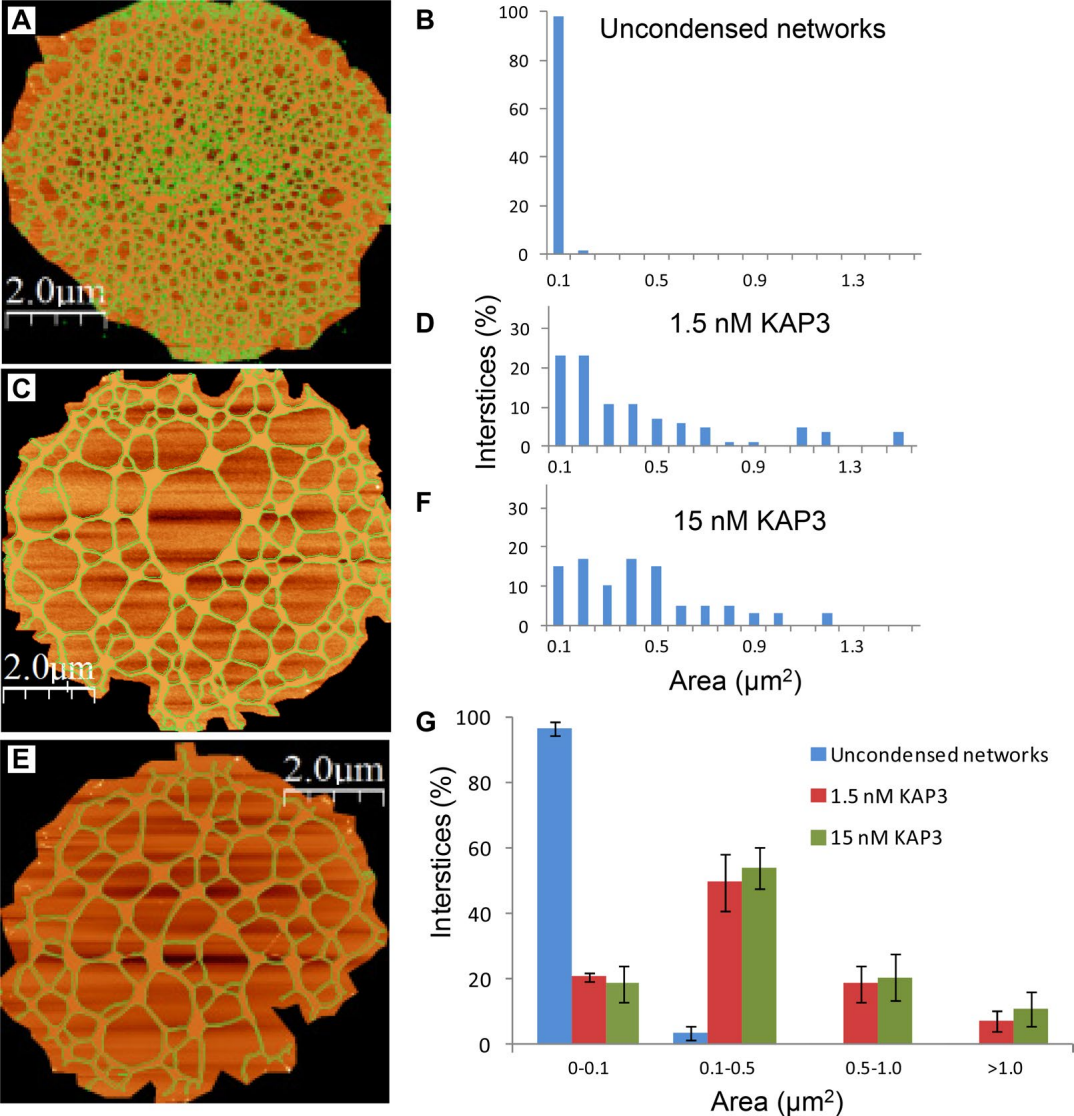
kDNA condensation intermediates consist of larger and less abundant interstices. **(A,C,E)** AFM scans of uncondensed kDNA and of networks that were interacted with 1.5 and 15 nM of KAP3, respectively. The network interstices are highlighted in green. **(B,D,F)** The size distribution of the network’s interstices and quantification of the area occupied by each interstices’ size-group in the networks, presented in **(A,C,E)**, respectively. Bars are 2 µm. **(G)** The average abundance of interstices (in percentage of the overall networks population) is plotted against KAP3 concentrations. Networks analyzed: uncondensed kDNA networks (n = 10) and condensation intermediate networks, generated in the presence of 1.5 nM (n = 6) and 15 nM KAP3 (n = 6). Standard deviation is indicated.

Figure 4A–C presents AFM images of uncondensed kDNA networks that were positioned on mica surfaces at different angles, demonstrating the folding of three-dimensional cup-like or basket-like structures into two-dimensional planar kDNA networks on the surface, as has been suggested previously based on electron microscopy. While scanning uncondensed kDNA networks under similar deposition conditions, we have observed images featuring at high resolution, rosettes structures, as described by Cavalcanti et al.^11,48^ (Fig. 4A–C). Notably, while previous electron microscopy analyses have reported that *C. fasciculata* kDNA forms an elliptical shape of approximately 10 × 15 µm^9^, our AFM analyses, revealed smaller dimensions of the network, of 6.0 ± 0.7 × 7.3 ± 0.5 µm (n = 22), similar to the values reported previously in AFM analyses of *C. fasciculata* kDNA networks^11,29^. An image of condensed kDNA network (Fig. 4D) and images at higher resolution of parts of the network (Fig. 4E,F), demonstrate the DNA condensation at the network’s foci. These images show that interactions of kDNA with a low concentration (1.5 nM) of KAP3 caused changes in the network organization and structure. First, new structures were observed in the AFM images, as structures protruding up from the nearly planar network level (see Figs. 4D–F and 5). These structures are distributed throughout the network. Concomitantly, the area of empty spaces (interstices) in the network increased dramatically (see Fig. 6, below), probably as a result of depletion of DNA circles from these areas and their piling in local condensation foci (herein designated LCF).

### kDNA condensation proceeds via assembly of fewer larger LCF and networks interstices

**Figure 7:**
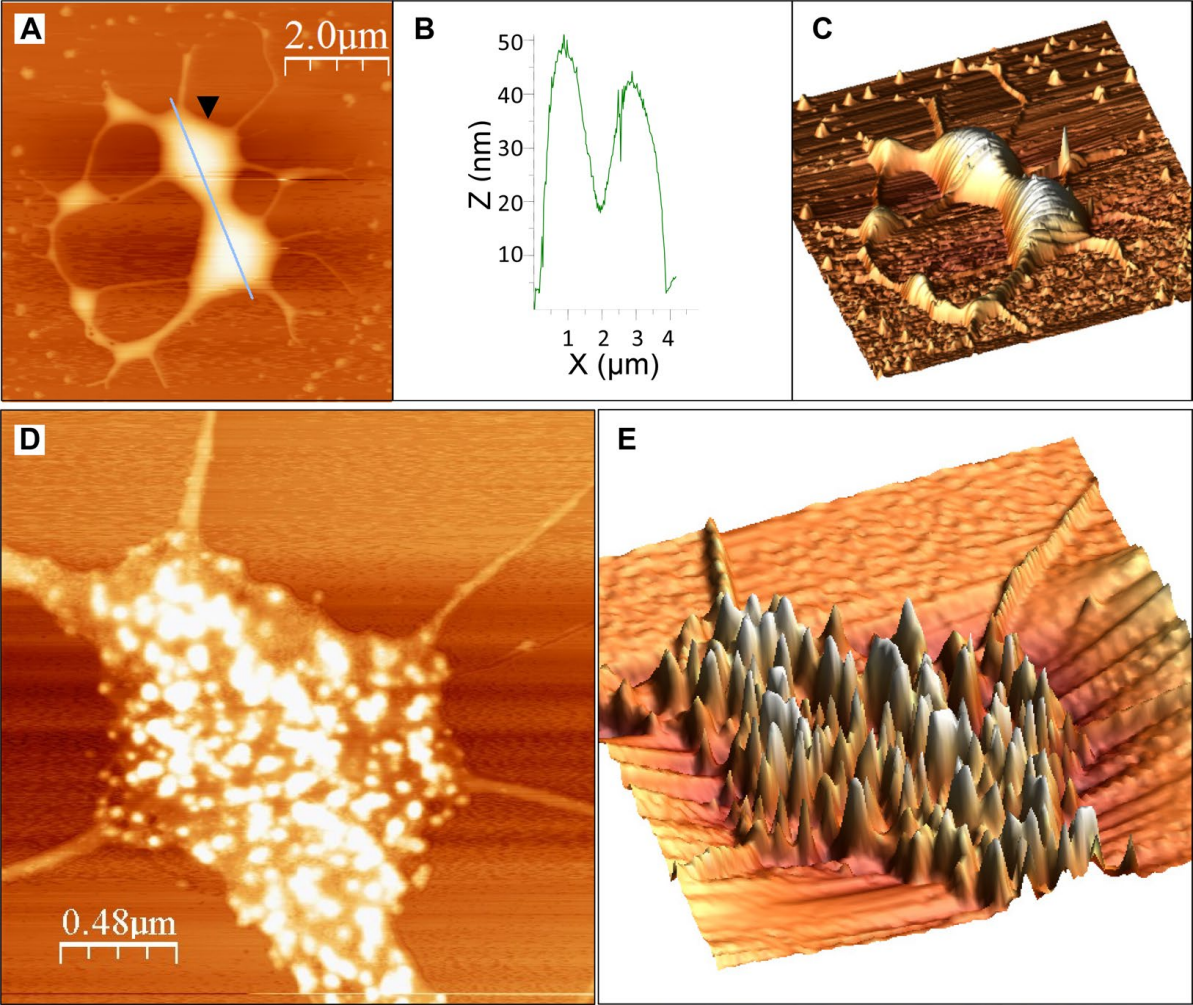
Hierarchy of kDNA condensation: LCF are assembled into few high order condensation centers. **(A)** 2D-display of an AFM scan presenting a kDNA network, which was interacted with 150 nM KAP3 (Bar = 2.0 µm). **(B)** A profile of the AFM scan (path is indicated by a line crossing the 2D image). **(C)** A 3D-display of the network presented in **(A)**. **(D)** Higher resolution scanning of one of the top condensation centers in the network, marked by an arrowhead in **(A)**. **(E)** A 3D-display of condensation center, marked by the arrowhead in **(A)**.

Two significant changes could be observed in the structure of the condensing kDNA networks. First, as shown in Figs. 5 and 6, the number of LCF in the condensing networks decreases and their size increases, in correlation with the increase in the concentration of KAP3 in the reaction mixture. No protruding structures higher than 2.4 nm could be detected in the 22 uncondensed networks analyzed (denoted by n = 22) (e.g., Fig. 5A). It has been previously reported that the height of double-stranded DNA measured in AFM images^11^ is 0.7 ± 0.2 nm^11,32^, which is lower than the ~ 2 nm diameter of crystallized duplex DNA obtained from X-ray analyses. Accordingly, the heights of the peaks observed here in the uncondensed network may correspond to DNA strands crossing over 2–3 times, as was also suggested by Cavalcanti et al.^11^. The height of LCF in the condensing kDNA networks, increases from an average height of 6.1 ± 0.9 nm above the mica plane in the presence of 1.5 nM KAP3 (n = 17) to 10.1 ± 1.2 nm (n = 11) in the presence of 15 nM KAP3 (Fig. 4J). It goes up to 50 nm in the presence of 150 nM KAP3 in the condensation reaction (see Fig. 7B). The 3D display (Fig. 5B,E,H) visualizes the overall increase in dimensions of the condensing foci in the kDNA condensation intermediates.

While no substantial changes were detected in the overall area occupied by kDNA networks, as the result of their interactions with low concentrations (1.5–15 nM) of KAP3, the number of the interstices in the network decreased, whereas the area occupied by the individual interstices increased considerably. Figure 6 describes examples of detailed analyses of the area occupied by each interstice in three individual networks: an uncondensed network (Fig. 6 A,B) and condensation intermediates, generated by interactions with 1.5 and 15 nM KAP3 (Fig. 6C,D,E,F, respectively). Further analyses of kDNA networks (Fig. 6G) revealed that in uncondensed networks, the most abundant interstices occupied an area at the range of 0.01–0.03 µm^2^ (~ 70% of the network area). Their area increases by more than an order of magnitude, with the most abundant interstices being at the range of 0.1–0.5 µm^2^ (~ 50% and ~ 55%) in the presence of 1.5 and 15 nM KAP3, respectively. In the presence of 15 nM a wider distribution of spaces is observed, with higher abundance of the 0.4–0.5 µm^2^ interstices (increasing from ~ 8% to ~ 12% in the presence of 1.5 nM and 15 nM KAP3, respectively; Fig. 6. Panels D–F with respect to panel B). The overall fraction of the networks area occupied by the network’s empty spaces increased from ~ 50% in uncondensed networks to ~ 70%, in the presence of 1.5 nM KAP3 and to ~ 75%, in the presence of 15 nM KAP3.

### Local minicircle foci assemble into higher order condensation structures

We measured 25 kDNA networks, condensed with 150 nM KAP3. With the increase in KAP3 concentration, the number of LCF in the network decreases, with a concomitant increase in the volume of the kDNA circles, piled in these LCF structures. AFM images of a condensing network, generated by the interaction of kDNA with 150 nM KAP3, is shown in Fig. 7. These images reveal a network, in which most of its content was condensed into few interconnected major condensation complexes (Fig. 7A–C). In one of them, we observed clear separation of the multiple foci, as shown in Fig. 7D–E. Remarkably, the scanning of an individual large condensation center at high resolution revealed multiple smaller condensation centers (Fig. 7D–E). It was very difficult to get clear imaging on top of most of the fully condensed networks, probably due to tip sticking and resulting instability in these imaging conditions.

Altogether, our observations suggest that kDNA condensation occurs via systematic assembly, in a hierarchical manner, of small LCFs into higher order condensation centers, which in turn, assemble into larger, higher order centers, until the several thousand DNA circles in the network form a single nucleoid complex.

## Discussion

We have previously reported^21^ on the specific interactions between the mitochondrial KAP3/4 proteins and UMSBP, which led to the remodeling of condensed kDNA networks. The specificity of these interactions was demonstrated in our previous studies^21^, following the remodeling of kDNA networks, which were condensed by human histone H1, versus networks condensed by KAP3/4^21^. Specificity of the remodeling reaction was further supported in the current study, by monitoring the UMSBP-mediated remodeling of kDNA networks, which were condensed by PLL. Unlike histone H1, PLL is a homo-polypeptide, which shares no characteristics with KAP3/4 proteins. The observation that UMSBP failed to decondense the PLL-condensed kDNA networks, even in the presence of two orders of magnitude molar excess of UMSBP (Fig. 2), is in accord with the notion that the UMSBP mediated remodeling reaction, is dependent on the capacity of UMSBP to interact specifically with the DNA-condensing agent.

Electron microscopy of kDNA, condensed in the mitochondrial matrix, suggests that kDNA is condensed in vivo into a disc-shaped nucleoid, in which minicircles are stretched-out and stand side by side, so that the height of the disc is approximately half the circumference of a minicircle (0.4 µm) and the disc consists of rows of minicircles^4^. In the current study we tried to understand the mechanism that converts the kDNA network from an uncondensed topological catenane, into a condensed mitochondrial nucleoid of a unique structure and organization. To address this question, we analyzed the structure of kDNA condensation intermediates by optical and AFM microscopy. The sigmoidal pattern observed in the plot of the condensed kDNA networks fraction versus KAP3 concentration suggests that KAP3-mediated condensation of kDNA may follow a cooperative kinetics (Fig. 3), as has been previously observed^13^. Models proposing mechanisms for cooperative binding of proteins to DNA have been discussed previously (e.g.^49–53^). Optical monitoring shows a clear change in the sigmoidal curve (Fig. 3) at 300 nM of KAP3, probably due to some saturation of the ratio of protein to DNA is achieved. Beyond that critical concentration, a smaller change in the KAP3 concentration was sufficient to promote the condensation more effectively. Although it is not possible to accurately interpret this change in the absence of direct kinetic monitoring, we speculate that it originates from a cooperative interaction between the network and KAP3.

Next, we questioned, how kDNA condensation, through interaction with KAP3, is affected by the network’s content of two distinct monomeric components, ~ 5000 DNA minicircles and ~ 25 maxicircles. It has been previously found that maxicircles form a topological network, distinct from that of the minicircles, within the kDNA network^25–27^. The two types of duplex DNA circles differ in their size, genetic function and mode of replication (reviewed in^1,4^). Yet, the two different catenanes are extensively interlocked with each other to form a composed kDNA network, or "network within a network" in *T. equiperdum*^25^. Fluorescence in situ hybridization (FISH) analyses, indicated that *C. fasciculata* maxicircles form approximately 8–10 foci within the kDNA disc^26^, while maxicircles in *T. brucei* networks spread throughout the kDNA disc, but are still linked topologically to each other^27^. This is nicely demonstrated in the post replication division of the network, when the minicircles are released from the network, the maxicircles are passively concentrated in the shrinking central zone of the network^54^. These observations support the notion that maxicircles are linked topologically to each other and to the minicircles, within the network, even though they may be scattered throughout the network in multiple foci. In exploring the effect of the network components on its condensation by KAP3, we speculated that at a low KAP3 concentration, most of the interactions of KAP3 would be with the highly abundant minicircle molecules (encompassing ~ 99.5% of the DNA circles in the network), while at increasing concentrations of KAP3, the frequency of KAP3-maxicircles interactions would increase. To study the effect of maxicircles on kDNA condensation, we have monitored the progress in condensation of maxicircles-deplete kDNA networks (minicircles-kDNA), upon interaction with increasing concentration of KAP3. The results, presented in Fig. 3, revealed a significant (P < 0.05) change in the progress of the condensation of minicircles-kDNA networks relative to that of native-kDNA networks, displaying a significantly higher increase in the abundance of condensed networks at low concentrations of KAP3. Remarkably, some saturation at the ratio of KAP3 to kDNA occurs here at a significantly lower critical concentration of KAP3 (100–300 nM), than that observed in native-kDNA networks. Beyond this critical point, we observed a sharp increase in condensation with saturation of the reaction at a significantly lower concentration of KAP3 than that observed with native-kDNA networks. Based on these observations, we suggest that the linkage between minicircles and maxicircles may stabilize the network structure, hence removal of maxicircles destabilizes the network, turning it into a more flexible and efficient substrate for condensation by the condensing protein.

The limitation of optical monitoring, which could detect changes in the structure of condensing kDNA networks, only at an advanced stage of condensation, directed us to analyze kDNA condensation intermediates, generated at the initial phase (in the presence of 1.5–150 nM KAP3) of the sigmoidal curve (see Fig. 3), using atomic force microscopy (AFM). Further analyses, using AFM, revealed the change in morphology through the generation of LCF in the kDNA condensing intermediates and its dynamics in detail (Fig. 4). Early kDNA condensation intermediates consist of interconnected condensation foci, probably mainly of minicircles, which are distributed throughout the network, forming an interconnected LCF network. We observed, unexpectedly, that at low concentrations of KAP3, the kDNA network did not shrink substantially over the whole area occupied by the network. Instead, within an apparently similar network perimeter, the empty spaces (interstices) area within the network increased considerably (Fig. 6), as a result of DNA circles piling into local foci forming the LCF structures, which grew in size in the presence of increasing concentrations of KAP3 (Fig. 5). We note that this phenomenon may be either, related to the in vivo condensation mechanism or to the interaction of the network with the surface that dictates condensation without shifting the LCF positions and therefore leads to increasing empty spaces.

Based on EM analysis, Chen et al.^6,7^, have described a 3D model, where the uncondensed kDNA network consists of several thousand DNA minicircles that are interlocked in a structure resembling the medieval chain mail armor, having an average valence of 3. The condensation of kDNA in the network can be explained in this 3D model, as such a network pleat readily, so that the rings in one row tilt in the opposite direction to the rings in the neighboring rows, yielding a remarkably flexible structure^7^. Our results fit well into the 3D chain mail armor model of Chen et al. We speculate that during the process of kDNA condensation, the flexibility of the chain mail network structure allows the interlocked minicircles in each row to slide one over the other, generating stacks of minicircles that yields the local condensation foci seen in the AFM images (Fig. 4). The foci of minicircles will, in turn, assemble in a hierarchical manner, into a higher order structure (Figs. 5 and 7), which finally yields a kDNA nucleoid assembly of a single layer of minicircles stacks, as the width of the disc is of one half the circumference of a stretched out minicircle (for illustrated model of the network organization, see Jensen and Englund (2012)^4^). One could speculate that given a diameter of 2 nm of the DNA double helix, the ~ 5000 minicircles positioned side by side, could form a row of a total length of 10 µm. Given a length of 1 µm of the disc axis (measured in electron micrographs of thin sections of kDNA in vivo), the ~ 5000 minicircles would occupy about 10 such rows (or stacks) of minicircles, where each stack could consist of approximately ~ 500 minicircles.

The condensation of kDNA network through a series of condensation intermediates, consisting of stacks of circles that assemble in a hierarchical manner, as suggested here, may reflect the basic mechanisms that mediate the condensation of this complex topological network into a remarkably organized nucleoid structure in vivo. The question, whether this unique nucleoid structure is determined by the particular characteristics of the condensing protein or rather by the specific constraints imposed by the topology of the catenane network has yet to be investigated.

## Methods

### Isolation and preparation of kDNA networks

*C. fasciculata* cell cultures were grown to a density of ~ 10^8^ cells/ml and purification of kDNA was carried out following Saucier et al.^55^, as modified for purification of *C. fasciculata* kDNA^56^, except that 0.7 mg/ml proteinase K was used, ultracentrifugation was conducted for 15 min, and ethidium bromide was extracted using 0.1xSSC-saturated iso-amyl alcohol. When further purification was required, kDNA was re-treated with proteinase K as above, followed by sequential phenol and chloroform extractions.

To prepare maxicircles-depleted kDNA networks (minicircles-kDNA networks), *C. fasciculata* isolated kDNA networks were incubated with restriction endonuclease *PstI*^28^ for 2 h, followed by a second addition of *PstI* and further incubation for 2 h. The kDNA networks were separated from the fragmented DNA by centrifugation (30 min, at 20,800 × *g*) and the pellet was washed twice (25 mM Tris–Cl pH 7.5). The extent of maxicircles depletion in *Pst1-*treated kDNA networks was quantified by quantitative PCR, using primers specific to the maxicircle Cox II gene as a template. Both maxicircles-depleted and native kDNA networks were analyzed. 563 bp fragments of the Cox II was amplified using forward primer 5′-TGAATGATATTTCTTATTGATGC-3′ and reverse primer 5′-ACATAATTCACTGCATTGACC-3′ (amplicon size 563 bp). Real time PCR reactions were performed in triplicates with the KAPA SYBR^®^ Fast Universal PCR master mix (Cat. No. KK4602). PCR reaction mixture (10 μl) contained 1 μl of each primer (10 pmol), 5 μl of SYBR Green and 1 ng of kDNA. Rotor-Gene Q (Qiagen) Thermo Cycler was used and PCR conditions were: 95 °C for 3 min for denaturation, followed by amplification of the target kDNA fragment for 40 cycles at 95 °C for 1 s, 59 °C for 20 s and 72 °C for 2 s. DNA was quantified using ImageJ software. A C_t_ difference = 6 was obtained, when Real time PCR was performed using undigested kDNA as a template (C_t_ = 11**)** versus *Pst1-*digested kDNA (C_t_ = 17**).**

### Preparation of *C. fasciculata* KAP3

The pET22b( +) plasmid, expressing *Cf*KAP3 protein was a generous gift of Prof. Dan S. Ray, from the Molecular Biology Institute and Department of Microbiology, Immunology and Molecular Genetics, at UCLA. Expression of *KAP3* in *E. coli* BL21, preparation of cell lysate, and affinity chromatography, using Ni–NTA beads (Qiagen), were performed as was previously described^57^.

### Analysis of kDNA condensation by fluorescence microscopy

kDNA condensation was examined as was previously described^21^. 200 ng (24.6 amol) of purified *C. fasciculata* kDNA networks (1 pM) incubated with *Cf* KAP3 as indicated, in a 25 µl reaction mixture containing 50 mM Tris–Cl pH 8.0 and 20 mM NaCl, for 5 min at 25 °C. A standard reaction, containing 45 pmol of KAP3 was used to completely condense the amount of kDNA, indicated above, and 90 pmol UMSBP were used to completely decondense the KAP3-condensed networks. The reaction mixture was adhered to poly l-lysine coated slides for 30 min in a humidity chamber and then mounted by Fluoroshield™ with DAPI (Sigma). It is noteworthy that since poly l-lysine can condense DNA, it may interfere with the accuracy and reproducibility of the quantitative fluorescence microscopy assay, due to its potential leakage from self-prepared poly l-lysine-covered slides. To avoid this potential interference, we have used commercially available poly l-lysine-coated slides (EMS, Cat. # 63410-01), which were proven to be suitable for this assay. Slides were examined in an Olympus IX71S8F inverted microscope, equipped with an Exi Blue™ Fast camera (Qimaging), with Apochromat oil immersion objective using × 100 magnification, controlled by CellSens Dimension 1.8 computer software (Olympus). Counting of stained networks was performed manually and included the average of 600 kDNA networks for each reaction.

### Characterization of kDNA condensation by atomic force microscopy (AFM)

A 3 µl-condensation reaction mixture, containing 50 mM Tris–Cl pH 8.0, 20 mM NaCl, 131 ng (16 amol) purified uncondensed kDNA (5.4 pM), and KAP3 protein as indicated, was incubated for 5 min at 25 °C. An equal volume of 25 mM MgCl_2_ was added and the reaction mixture was placed on a freshly cleaved mica surface. The sample was incubated for 1 min at room temperature, rinsed gently in double distilled water and dried under a nitrogen flow. The samples were scanned in dynamic mode using AIST-NT SmartSPM™ 1000 in ambient. Samples were measured using soft cantilevers (OMCL-RC800PSA, Olympus Optical Co., Ltd) of nominal force constant 0.3 N/m, resonance frequency of 67–69 kHz and tip radius 15–20 nm. The images were analyzed using WSxM Probe Microscope software^58^ (Nanotec Electronica S.L). Three-dimensional (3D) displays of AFM scans were based on the total data collected in the scan. Height profiles in AFM scans, were based on measurements corresponding to the random paths, indicated by the lines crossing the images.

## Data Availability

All data generated or analyzed during this study are included in this published article.
